# Medical and Physical Intelligence

**Published:** 1803-07-01

**Authors:** 


### MEDICAL AND PHYSICAL INTELLIGENCE.

### [ FOREIGN AND DOMESTIC. ]

The VACCINE INOCULATION is fully established throughout all the British Presidences in India. Dr. JENNER has been informed, that at Madras, the children of every European in that settlement have been already inoculated. From Bengal he also learns, that the Hindoos receive it with the greatest ardor, from the veneration these people pay to the cow, as well as for the security they find in it from the Small-pox.

Dr. JENNER has also received a very curious and interesting piece of intelligence from Dr. SACCO, of Milan, whose elegant work on Vaccine Inoculation is well known. Dr. Sacco was among those who not only doubted the validity of Dr. Jenner's opinion of the origin of the Cow-pox, but imagined he was in possession of facts sufficient to overthrow it completely. However, a singular circumstance happened, to convince the Doctor of the fallacy of his former observations. One of his coach-horses had the common malady of the heel; his coachman attended to the sore, and, in consequence, exhibited several pustules upon his hands, with all the characteristic marks of those derived from the cow. It was deemed too late to take matter from them for Inoculation; but soon after this, a similar case occurred on the fingers of the coachman of another gentleman in Milan. From these pustules Dr. Sacco inoculated nine children and a cow. Three of the children were infected, and had the disease exactly in the same way as if it had been communicated from the cow. With matter taken from these children, other inoculations were performed; and at the time this account was transmitted, it had reproduced itself correctly a fourth time. Dr. Sacco also adds, that he has inoculated six other children with the matter of grease, and on two of them it produced pustules with all the genuine characters of the Vaccine. He intends giving a coloured plate of the disease in the foot of the horse.

M. Basse gives the following as the best method of preparing muriatic æther with the simple acid:—Melt marine-salt in a crucible, and keep it in fusion an hour, or till the whole of the water of crystallization be dissipated; put twenty ounces of this salt into a tubulated retort, adapt to it a curved tube, and plunge the tube to the bottom of a bottle with two necks, into which have been poured ten ounces of alkohol prepared by mixing, in a retort, three parts of highly rectified spirit of wine with one of potash, melted and pulverised whilst hot, and distilled till it is diminished one-half. When the whole is well luted, pour into the retort, in very small quantities at a time, ten ounces of highly concentrated sulphuric acid. After each introduction of acid, close the tube carefully, and put in no more acid till the salt has ceased bubbling. The cork of the other neck of the bottle must be taken out from time to time, to suffer the air condensed above the alkohol to escape. After the acid is introduced, place the retort on a sand-bath, and heat it gradually, till all the muriatic acid be expelled. During this part of the operation, care must be taken frequently to cool the bottle containing the alkohol, by wrapping a wet cloth round it. The alkohol thus charged with acid, is then put into a retort, and distilled to one-half; shake the distilled liquid with alkaline-ley, to carry off the acid, decant the æther which is found on the surface, and keep it in bottles well corked. From the above-mentioned quantities two ounces and a half of æther are usually obtained.

---

Mr. Buchholz, of Erfurt, has made a series of experiments, in order to ascertain the existence of cobaltic acid, or whether the metal of cobalt is really capable of being changed into an acid by its combination with oxygen, as has been asserted by Signor Brugnatelli, on whose authority the cobaltic acid was adopted. The results of these interesting experiments, which proved to be a negative of this doctrine, are the following:

1. Cobalt is not to be changed into the state of an acid, by treating it with any kind of nitric acid, or according to any known method.

2. It is likewise not to be effected, according to Mr. Scheele's method, by means of muriatic and nitric acid; the former having a greater affinity to oxygen than cobalt.

3. The supposed cobaltic acid of Signor Brugnatelli is most probably nothing but a disguised arsenic acid, or a combination of arsenic acid with ammonia and some oxyd of cobalt; at least such a composition produced the same phenomena, which Brugnatelli considers as characteristics of his cobaltic acid.

4. On treating cobalt, or oxyd of cobalt, which is entirely free from arsenic, with nitric acid and ammonia, no such combination can be obtained, possessed of the characteristics ascribed by the Italian chemist to his cobaltic acid.

5. These experiments may serve to prove how necessary it is to refer any new discovery of this kind to the test of repeated experiments, before it is admitted as certain.

---

Mr. Tromsdorff gives the following method for obtaining metallic cobalt perfectly pure. "Mix a pound of best saffre with four ounces of nitrat of potash, and two ounces of pulverized charcoal, and throw the mixture in small portions into a red-hot crucible; repeat the same operation three times; at the third time leave the matter exposed to a white heat; remove it rapidly, and add four ounces of black flux: place the crucible in the furnace, and let it remain perfectly red-hot for an hour; when cold, separate the reduced part of the cobalt, which, in consequence of the treatment to which it has been subjected, has lost great part of its arsenic and iron; it must then be mixed with thrice its weight of nitrat of potash, and the mixture deflagrated in small portions in a red-hot crucible. By this last operation the iron is completely oxydated, the arsenic is converted into acid, and taken into combination by the potash. By levigation with warm water, all the saline parts are carried off, and the oxyd of cobalt is separated by the filtre. The oxyd is to be dissolved in a suitable nitric acid, and the solution filtered."

---

Cit. Delunel has contradicted the opinion of those, who think the distilled waters of inodorous plants useless in medicine, and not differing from common distilled water; and he relates two instances, in order to prove his assertion. The distilled water of borage, (borago officinalis) on being evaporated, obtained an orange colour, and the residuum had a peculiar smell, like that of artichokes. In another experiment, the residuum became of a straw yellow colour, and it was precipitated by ammonia. On suffering the water to stand for about a month in a bottle that was well shut, a strong smell of hepatic gas was perceived on opening the bottle; whence he thinks it may be used as a mineral water. The water of solanum nigrum, L. or common night shade, emitted the smell of opium, but disappeared on the evaporation being continued. The red extract contained a considerable portion of nitrat of kali.

---

Mr. Schrader, Apothecary at Berlin, has made the interesting discovery, that the Prussic acid is contained in the aqua lauroce-rasi, and the distilled water of the flowers of the peach-tree, as likewise in the infusion of bitter almonds. He was led to this discovery by observing, that the Prussic acid has the quality common with those distilled waters and infusions, of killing animals; and that the Prussic acid, as well as the abovementioned water, possesses the smell of bitter almonds. This discovery is likely to be very advantageous to medicine as well as to the arts; and the author thinks it probable, that what is hitherto called the narcotic principle ciple may be nothing but the Prussic acid; and in order to prove this conjecture, he intends making experiments with opium, hyosciamus, belladonna, and the other narcotics.

---

The new metal which has been announced to the public, under the name of *palladium*, is found to be a composition of two parts of platina and one of mercury.

---

Mr. W. HENRY has published a third edition of his excellent Epitome of Chemistry, revised and considerably enlarged.

---

Dr. PERCIVAL, of Manchester, has in the press, and nearly ready for delivery, a work which will comprehend a General System of Medical Ethics. The outline was sketched several years ago, and constitutes the code of regulations by which the Faculty of the Manchester Infirmary agreed to regulate their official conduct, and their intercourse with each other. But the extended work will be addressed to the Medical Profession at large; and will include also the duties of such of its members as are unconnected with public establishments of charity.—The aphoristic form has been chosen, as the best calculated to define with precision those principles of urbanity and rectitude which should govern the conduct of the members of the medical profession to their patients and to each other. Copious Notes and Illustrations will form an Appendix, and will complete the plan of the undertaking.

---

Mr. DEWAR has a work in the press, which will be published in a few weeks, entitled, "Observations on Diarrhœa and Dysentery, as these Diseases appeared in the British Army, during the Campaign in Egypt in 1801. To which are prefixed, A Description of the Climate of Egypt, and a Sketch of the Medical History of the Campaign."

